# Supplementary material for: Kemeny Constant-Based Optimization of Network Clustering Using Graph Neural Networks
Source: J Phys Chem B. 2024 Aug 15;128(34):8103–15. doi: 10.1021/acs.jpcb.3c08213 (PMC11367579; doi:10.1021/acs.jpcb.3c08213)
Supplement: Supplementary file 1 — jp3c08213_si_001.pdf [file jp3c08213_si_001.pdf]

Kemeny constant-based optimization of network clustering using Graph Neural Networks
Supporting Information

Sam Alexander Martino[1], João Morado[1], Chenghao Li[1], Zhenghao Lu[1], and Edina Rosta[1*]

[1]Department of Physics and Astronomy, University College London
[*]Correspondence: e.rosta@ucl.ac.uk

SI(A) FEATURIZATION

In order learn effectively, GNNs require a distinct feature vector F_i associated with each node N, which is used as the initial input to the model. There are several different ways to produce N feature vectors of dimension d_{feat}. Here, we aimed to use general features based on the properties of the MSM, and did not include system specific information, such as structural properties, which could also be used for molecular MCs. Below is a list of the different featurization methods we used for KC-based clustering.

- **Markov/Rate Matrix:**
  As a MC can be fully defined by either a Markov matrix $\mathbf{Q}$ or rate matrix $\mathbf{K}$, both of shape $(N \times N)$, the simplest featurization is to use the columns of either defining matrix corresponding to the outgoing rates/transition probabilities as the N feature vectors for each node with $d_{\text{feat}} = N$. This produces a set of features for each node $\mathbf{F_i}$ where each element of the feature matrix is simply a column of the Markov or rate matrix: $\mathbf{F_i} = (Q_{1i}, ..., Q_{Ni})$, or analogously, $\mathbf{F_i} = (K_{1i}, ..., K_{Ni})$.
  We also tested whether performing PCA on the matrix $\mathbf{K}$ or $\mathbf{Q}$ prior to splitting into individual F_i would improve performance, as done in other work on clustering using GNNs [2]. To do so, we perform PCA using the Sci-Kit Learn Python library [1,4]. Interpreting each column $\mathbf{F}_i$ as a sample and the rows as features, we take all components of the PCA. This can be seen as a linear rotation of the defining matrix onto the perpendicular directions along which the node transition probabilities or rates vary the most, with the aim of making nodes with similar transitions appear more similar.

- **Adjacency Matrix:**
  As in the main work, we interpret the adjacency matrix of an MC to be a simple cutoff of the defining matrix $(Q_{ij} + Q_{ji})/2 > c = 0.015$. This yields a binary $(N \times N)$ square matrix, which we split into columns representing each node with $d_{\text{feat}} = N$. We also again tested a PCA of this, utilizing the same strategy as before.

- **MFPT Matrix:**
  As the MFPTs are one of the most relevant properties for the KC, we tested using them as node features. Firstly, the MFPT matrix with elements t_{ji} of the unclustered graph was calculated using Eq. (12). This generates an $(N \times N)$ matrix of the MFPTs between nodes in the network. If needed, we can then extract the PCA features, before finally splitting the matrix up into feature vectors F_i of size $d_{\text{feat}} = N$ using the columns of the MFPT matrix.

- **Eigenvectors:**
  As the eigenvalues and eigenvectors of the Markov matrix describe important kinetic properties, we also used the eigenvectors of the Markov or rate matrices as feature vectors.

- **Trainable Embedding:** Trainable embeddings have found much success in Large Language Models (LLMs), where they provide an efficient way to represent the sentiments of different tokens, and have been employed previously as random features for GNNs. Trainable embeddings are equivalent to an associative array data structure, where a predefined set of keys each point to a vector of arbitrary size. The vector is initialized with random numbers which are then trained along with the rest of the network during back-propagation steps. We used the torch implementation [3] , which uses integer keys from $\{1 \ldots N\}$ representing each node. Several embedding sizes were tested before settling on $d_{\text{feat}} = 128$ which provided the best performance across all systems.

- **Single layer linear network:**
  A single feedforward layer that takes as input for each node the $3N$-dimensional vector that is formed by concatenating the corresponding columns of the (i) Markov/rate, (ii) adjacency, and (iii) MFPT matrices. The output dimension was $d_{\text{feat}} = 128$ in all systems, and the weights of this layer were updated as part of the training process.

SI(B) ADDITIONAL FIGURES

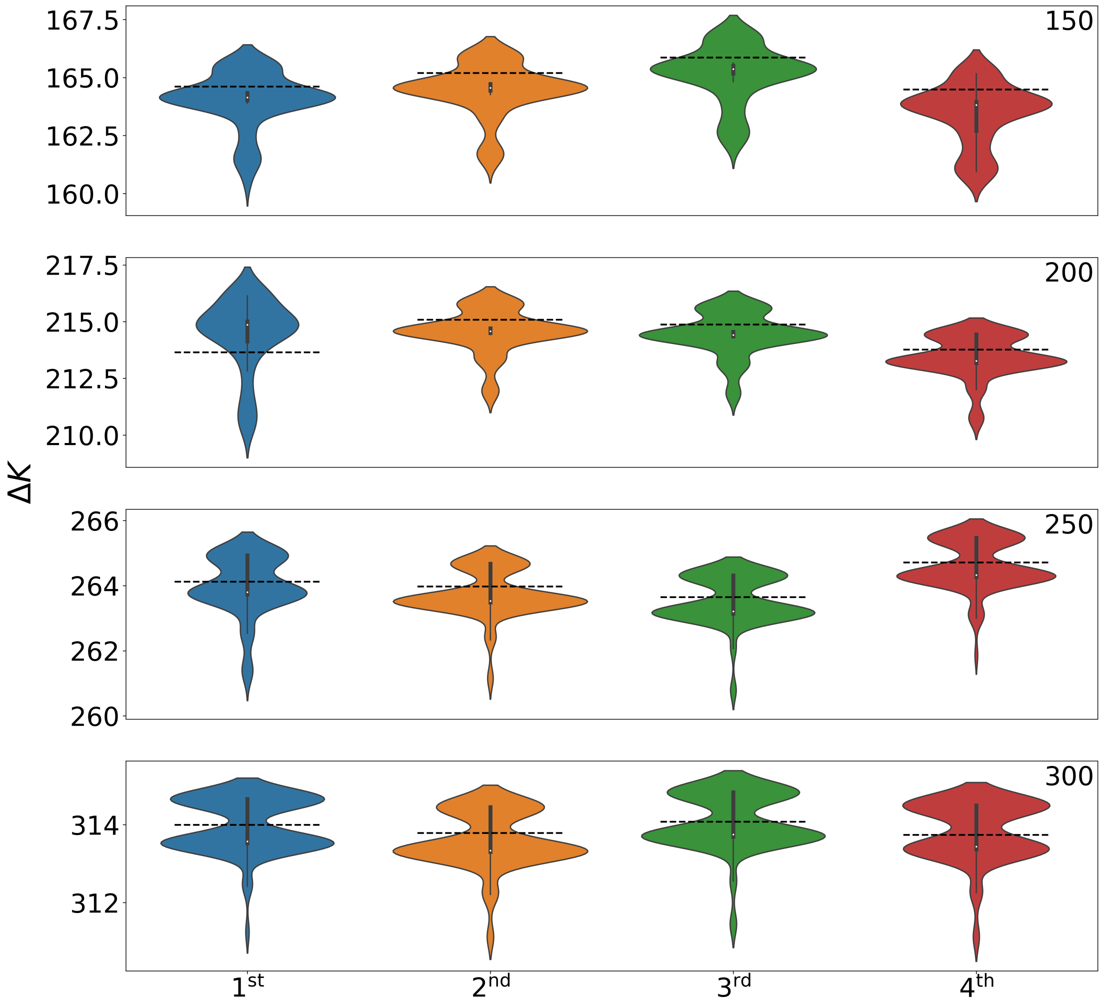

Figure S1. A comparison of the ΔK values of the partitionings found by PCCA+ (the dotted black line) and the GraphSAGE + LD GNN method on four SBM networks, where the GNN provides a clustering with the most optimal value. These four networks represent the largest difference in our examples between the Kemeny optimum and the ΔK value corresponding to the PCCA+ clustering.

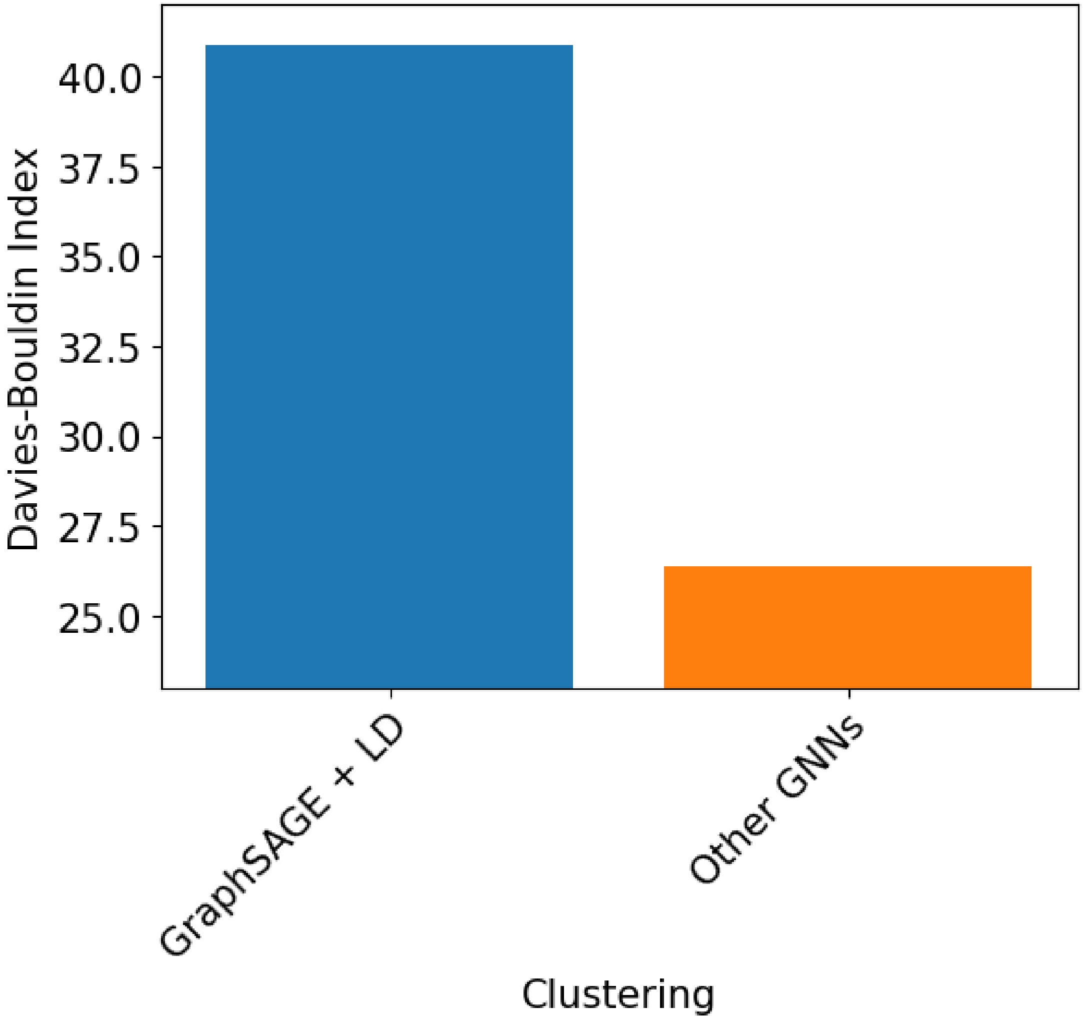

Figure S2. DBI values corresponding to clusterings found for the 1D example.

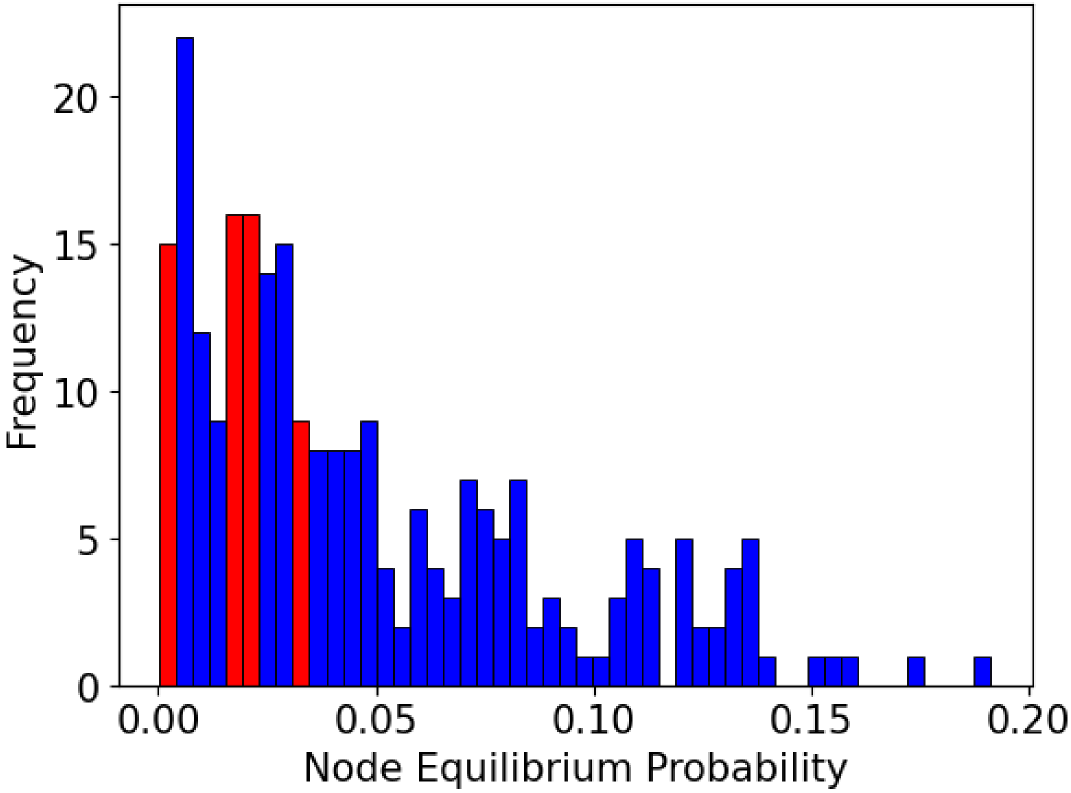

Figure S3. A histogram of the equilibrium probability distributions of each node in the pentapeptide example. Red bars indicate the presence of a node which is placed into a different cluster by PCCA+ and the GraphSAGE + LD method. These differing nodes represent 7.4% of the overall node populations.

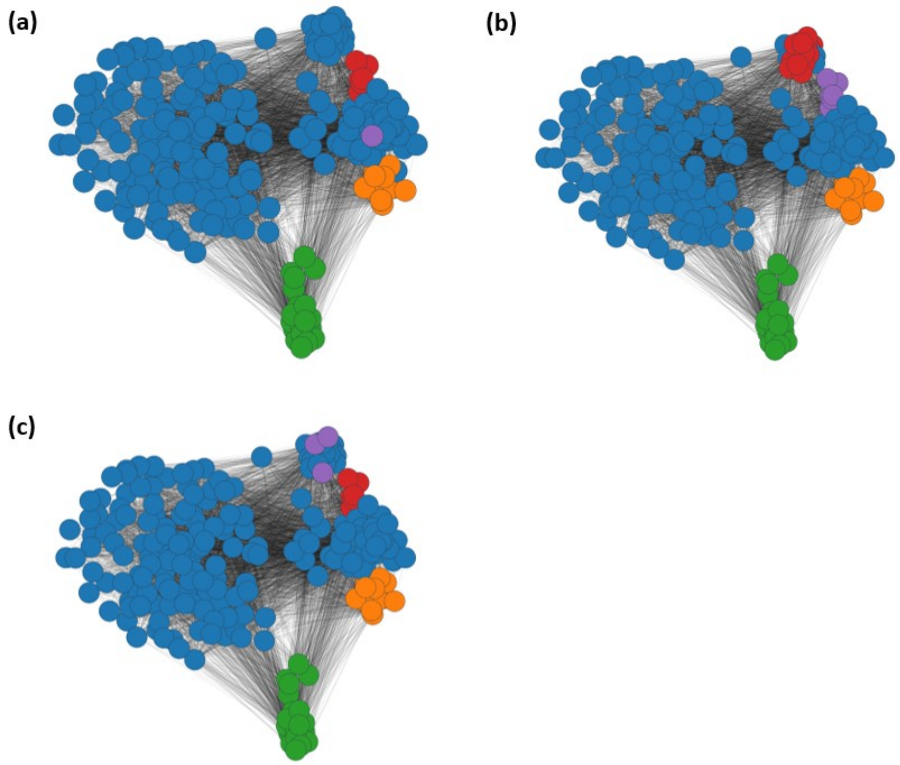

Figure S4. A comparison of the pentapeptide network partitionings with the lowest ΔK value found by each method used. Figures **(a)**, **(b)**, **(c)** show those found by the GATv2 + TD, GraphSAGE + TD, and GATv2 + LD respectively

REFERENCES

[1] PCA, SciKit-Learn 1.5.0 Documentation. `https://scikit-learn.org/stable/modules/generated/sklearn.decomposition.PCA.html` (accessed June 21, 2024).

[2] Nazi, A., Hang, W., Goldie, A., Ravi, S., and Mirhoseini, A. (2019). A deep learning framework for graph partitioning. *ICLR*.

[3] Paszke, A., Gross, S., Massa, F., Lerer, A., Bradbury, J., Chanan, G., Killeen, T., Lin, Z., Gimelshein, N., Antiga, L., et al. (2019). Pytorch: An imperative style, high-performance deep learning library. *Advances in neural information processing systems*, 32.

[4] Pedregosa, F., Varoquaux, G., Gramfort, A., Michel, V., Thirion, B., Grisel, O., Blondel, M., Prettenhofer, P., Weiss, R., Dubourg, V., et al. (2011). Scikit-learn: Machine learning in python. *Journal of machine learning research*, 12(Oct):2825–2830.
